# Management of male erectile dysfunction: From the past to the future

**DOI:** 10.3389/fendo.2023.1148834

**Published:** 2023-02-27

**Authors:** Chang-Ming Wang, Bao-Rui Wu, Ping Xiang, Jun Xiao, Xue-Chun Hu

**Affiliations:** Department of Urology, The First Affiliated Hospital of USTC, Division of Life Sciences and Medicine, University of Science and Technology of China, Hefei, China

**Keywords:** erectile dysfunction, phosphodiesterase 5 inhibitor, intracavernosal injection, hormonal replacement therapy, vacuum erection device, penile prosthesis implantation, low-intensity extracorporeal shock wave, stem cell injection therapy

## Abstract

Erectile dysfunction is a common disease of the male reproductive system, which seriously affects the life quality of patients and their partners. At present, erectile dysfunction is considered as a social-psychological-physiological disease with complex etiology and various treatment methods. Oral PDE5I is the first-line treatment for erectile dysfunction with the advantages of high safety, good effect and non-invasiveness. But intracavernosal injection, hormonal replacement therapy, vacuum erection device, penile prosthesis implantation can also be alternative treatments for patients have organic erectile dysfunction or tolerance to PDE5I. With the rapid development of technologies, some new methods, such as low-intensity extracorporeal shock wave and stem cell injection therapy can even repair the organic damage of the corpora cavernosa. These are important directions for the treatment of male erectile dysfunction in the future. In this mini-review, we will introduce these therapies in detail.

## Introduction

Erectile dysfunction (ED) is defined as the consistent inability to attain and maintain an erection sufficient to perform satisfactory sexual intercourse (1). ED is a common male problem at all ages that has a great impact on the quality of life of sufferers and their partners. More than 150 million men worldwide are reported to have ED in different extent (2). Due to racial and regional differences and different definitions of ED, there is a large gap in the existing epidemiological data of ED. In the United States, the incidence of ED is 25.9 cases per 1000 people, and it increases with age with more than 70% of men over 70 years old affected by ED. It is predicted that by 2025, 322 million men worldwide will have ED (3–5). Studies have shown that the occurrence of ED is associated with many comorbidities and risk factors, such as aging, smoking, obesity, decreased androgen levels, cardiovascular disease, depression, prostate surgery, and penile trauma (6–8).

**Figure 1:**
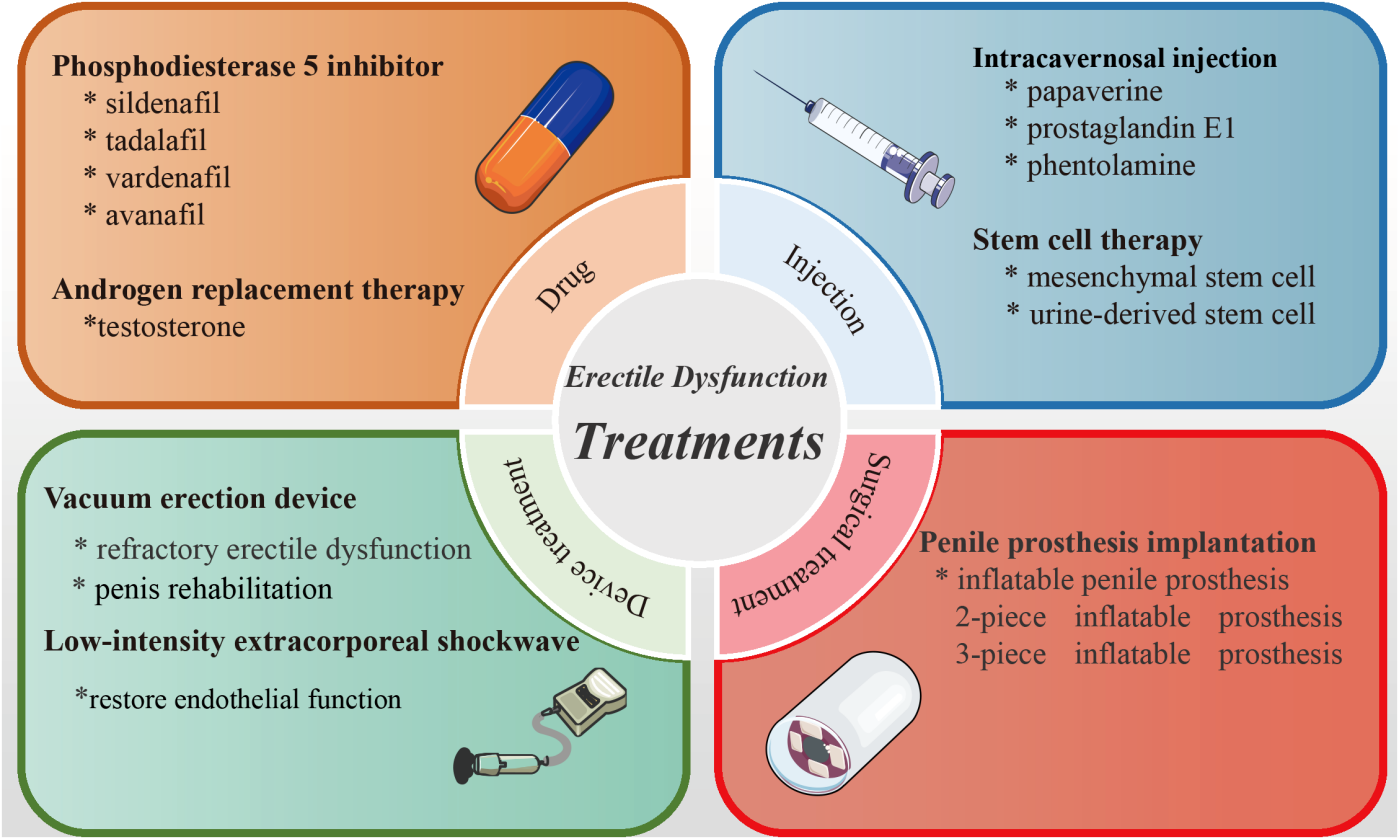
Summary diagram of the therapies of erectile dysfunction.

Normal penile erection is a neurovascular phenomenon controlled by psychological factors and coordinated by endocrine, vascular, and nervous system (9). The first step in management of ED is often making lifestyle changes, such as losing weight, reducing alcohol intake, and avoiding smoking. These psychosocial methods are effective when ED is mainly caused by emotional or psychological factors (10). Current therapies to treat ED mainly include oral phosphodiesterase 5 inhibitor (PDE5I), intracavernosal injection, hormonal replacement therapy, vacuum erection device, penile prosthesis, low-intensity extracorporeal shock wave (Li-ESW), and stem cell injection therapy (11) (**Figure 1**). Under different conditions, any option can be the first line of treatment. To data, PDE5I is still the most popular treatment option due to its good efficacy, safety, and non-invasiveness. But, the growing number of patients with no or low response to PDE5I, and the potential of adverse reactions have prompted the development of safer and more effective treatments (12). In this paper, we will introduce various treatment methods for ED in detail based on current research progress.

## Drug therapy: Phosphodiesterase 5 inhibitors

Phosphodiesterase 5 (PDE5) is highly expressed in vascular smooth muscle and it is the most common PDE subtype in penile smooth muscle. Penile erection mainly depends on the activation of NO/cGMP signal pathway and the function of PDE5 is to block the decomposition of cyclic guanosine monophosphate (cGMP) (13, 14). the NO produced by non-adrenergic/non-cholinergic neurons and endothelial cells is released into the corpora cavernosa, resulting in an increase in the concentration of cGMP, thus promoting the relaxation of smooth muscle in the corpora cavernosa and the expansion of penile blood vessels, finally leading to vascular filling and penile erection (15). Therefore, PDE5I can enhance erectile response and treat erectile dysfunction by enhancing the downstream cGMP effect caused by NO.

PDE5Is are the first-line treatment for erectile dysfunction (16). At present, four PDE5I drugs have been approved by the FDA, namely sildenafil, tadalafil, vardenafil, and avanafil. These PDE5Is have different pharmacokinetic properties but have similar efficacy, safety, and tolerability (17). Among them, sildenafil is the first approved, safe, and effective oral drug for the treatment of ED. In a randomized, double-blind study, Goldstein et al. (18) included 532 male patients diagnosed with ED at least 6 months and randomly assigned 316 patients to the sildenafil group (25, 50 or 100ng) and 216 patients to the placebo control group. After 24 weeks, the erectile function of the oral sildenafil treatment group improved significantly, and men taking 100 mg of sildenafil showed a better therapeutic effect compared to the placebo control group. Sildenafil can also be used in combination with other drugs. In another randomized controlled study, 59 patients with organic ED were included in the study (19). One group received oral sildenafil 50 mg, the other group received oral sildenafil 50 mg and L-arginine 1g, after 8 weeks of treatment, patients in the combined treatment group showed better erectile function. These studies have proved the effectiveness of sildenafil in the treatment of ED and its potential in combination with other therapies. As a powerful and highly selective PDE5I, avanafil has been reported to have better effects and fewer adverse reactions. Kumar et al. (20) recently reported a randomized, controlled, double-blind clinical trial in which 220 patients with ED were randomly divided into two groups in a 1:1 ratio, they were given orally 100 mg of avanafil and 50 mg of sildenafil respectively. International index of erectile function (IIEF) score, sexual encounter profile (SEF), and adverse drug events were evaluated, and the results demonstrated that avanafil took effect quickly, and most people showed good erectile function after 15 minutes of medication.

In addition to acting on PDE5 of cavernous smooth muscle, PDE5I also inhibits PDE5 and its isozymes in blood vessels, viscera, skeletal muscle, platelets, and other tissues, causing reactions in multiple systems. During treatment, adverse reactions such as headache, blush, dyspepsia, and visual disturbance can occur (21). In addition, after taking tadalafil for 6 months, the weight of testis, sperm quantity and sperm activity of the aged male rats were significantly reduced (22), after 12 weeks of oral administration of sildenafil, tadalafil, and vardenafil in male rabbits, the number of sperm in sperm cells and testis were also decreased (23). In general, PDE5I is a safe, effective, and well-tolerated first-line treatment for ED, for most patients, 50 mg sildenafil is the preferred treatment, after drug tolerance, 10 mg tadalafil or 100 mg udenafil can be used instead (24). Not only that, but current studies also found new molecular mechanisms of ED beyond the ‘NO/cGMP’ pathway. For example, hyperglycemia and increased oxidative stress are main contributors to endothelial dysfunction in ED patients complicated with diabetes mellitus (25). The major regulatory unit of myosin light chain phosphatase MYPT1 regulated ED by G-protein couple receptor pathway, and the lotusine could recover the level of MYPT1 and improve the function of injured penile smooth muscles. These studies provide novel therapeutic targets for the treatment of ED in the future (26).

## Intracavernosal injection

Intracavernosal injection (ICI) of vasoactive drugs such as papaverine and prostaglandin E1 to induce penis erection is a breakthrough in the treatment of ED and can also be used as a diagnostic method (27). ICI is an effective local drug therapy for ED, individualized treatment plans can be formulated according to the individual conditions and needs of the patients (28). The combination of different vasoactive drugs and different injection doses can significantly improve the treatment effect and reduce complications. The study showed that the patients injected with papaverine and prostaglandin E1 could achieve satisfactory erectile function, which was better than patients injected with prostaglandin E1 alone (29). In a retrospective study, the researcher included 105 middle-aged and elderly patients and found that after ICI treatment, the patient’s penis hardness increased, erectile function improved, and there were no obvious complications, this means that ICI therapy is safe and feasible (30). However, with the use of PDE5Is, the clinical application of ICI has gradually decreased, because it has a high dropout rate and is related to priapism, ecchymoses, hematoma, and penile fibrosis (31). At present, the combination of ICI and Doppler ultrasound is mainly used in the diagnosis of ED and the evaluation of penile hemodynamics (32).

## Hormonal replacement therapy

Androgen plays an important role in promoting the normal growth of the penis and stimulating the secondary sexual characteristics of men. Androgen is mainly secreted by the testis, androgen deficiency will lead to a series of pathophysiological conditions, which will damage the sexual function and overall health of the body (33). Researchers found that serum total testosterone, especially free testosterone and bioavailable testosterone levels of men will gradually decrease with age. A study showed that 64% of men over 40 years of age will be diagnosed with moderate, severe and very serious ED and older men over 60 years of age are more likely to suffer from more serious ED (34). Similarly, Rabijewski et al. found that 53% of the elderly men over 65 years old had lower testosterone levels, and ED was more serious in these men. They also found that there was significant negative correlation between age and testosterone (r=-0.3328, p<0.05), IIEF score and testosterone (r=-0.3149, p<0.05), and age and IIEF score (r=-0.3463, p<0.05) (35).

In clinic, androgen replacement therapy can restore the serum testosterone level to normal, and improve the sexual desire of patients with hypogonadism. In addition, compared with the placebo group, after receiving androgen replacement therapy, the patients would get better mood and the depression was relieved (36). Another study showed that in older men with low testosterone levels older than 65 years, the frequency of sexual activity increased significantly and the sexual desire improved after one year of androgen replacement treatment (37). More importantly, androgen replacement therapy combined with PDE5I can effectively treat ED, and patients’ erectile function can even be maintained well after drugs withdrawal (38).

## Vacuum erection device

Vacuum erection device (VED) is a mechanical device that can increase the blood flowing into the corpora cavernosa by creating a vacuum environment of up to 250 mmHg and there is a restraining ring at the root of the penis to maintain congestion, promote erection (39). VED is mainly used to treat patients with organic ED and it has high success rate and small side effects (40). In a recent study, 56 middle-aged and elderly patients with ED were treated with VED, 96% of the patients believed that the device could promote the ability of erection and 94% of the patients and their partners thought that they regained satisfactory sexual activities after VED treatment. Nevertheless, nearly 28.6% of the patients reported physical discomfort when using the device, usually due to the pain caused by using the restraining ring (41).

In addition to treating ED, VED can also treat penis atrophy after radical prostatectomy by enhancing oxygen saturation in the corpora cavernosa (42). Rats with penile atrophy and decreased erectile function were created by bilateral cavernous compression injury (BCNI). After 6 weeks of treatment with VED for BCNI rats, the penis diameter of the rats was increased, the degree of atrophy decreased, and the oxygen saturation in the corpora cavernosa increased. These results show that VED treatment has therapeutic effect by increasing the anti-hypoxia ability of corpora cavernosa (43). PDE5I tolerance is a common outcome of oral medication in ED patients and about 30% of ED patients have no obvious response to PDE5I treatment eventually (44). After using VED treatment for these patients, their erectile function improved and their sexual desire increased. VED is the second-line treatment for ED, but it should be considered as the first-line treatment for some men who have tolerance to PDE5I or need penis rehabilitation (45).

## Penile prosthesis implantation

Penile prosthesis implantation (PPI) is currently the third-line treatment for ED. Because it can cause irreparable damage to the smooth muscle of the corpora cavernosa, it is usually considered when oral PDE5I drugs, intracavernosal injection, and VED therapy are ineffective (46). 3-piece inflatable prosthesis is the most common implant at present, and is also the most satisfactory (47). The 3-piece inflatable prosthesis can manually adjust the thickness, length, and hardness of the penis, and simulate the natural erection process. Therefore, it should be recommended for patients who choose PPI.

An early multi-center study reported that more than 90% of patients with ED and their partners can achieve normal sexual activity after receiving penis prosthesis implantation (48). Recent study has shown that penis prosthesis implantation is particularly suitable for ED patients secondary to Peyronie’s disease (49). Nevertheless, PPI is costly, traumatic, and prone to complications, such as prosthetic infection, pump migration, automatic inflation, secondary surgery, etc. This is the main reason why it cannot become a first-line treatment (50).

## Low-intensity extracorporeal shock wave

Low-intensity extracorporeal shock wave (Li-ESW) is a physical shock wave that emits energy density lower than 0.1mj/mm^2^. As a non-invasive treatment technology, Li-ESW focuses on the target tissue area through the sound wave passing through the tissue structure (51). Studies have shown that one of the causes of ED is the decreased blood circulation in the corpora cavernosa, Li-ESW can stimulate the expression of eNOS, VEGF and other vascular growth factors in the corpora cavernosa, expand blood vessels, induce neovascularization, promote blood flow, and improve erectile function (52, 53).

Vardi et al. (54) treated 20 middle-aged patients with vascular ED with Li-ESW, the patients received 12 Li-ESW treatments within 6 weeks. The results showed that the erectile function of 75% of patients was significantly improved, and the IIEF score was obviously increased, the penile blood flow, erection duration and penile hardness of the patients were also increased. Li-ESW therapy has also been proved to be a safe and effective method for those patients with poor effect of PDE5I (55). Another study found that the use of Li-ESW can reverse the PDE5I tolerance, and more than 50% of the patients can achieve sufficient erectile stiffness (56).

Li-ESW is a promising treatment for refractory ED. It can restore the endothelial function of the penis and increase the blood flow of the corpora cavernosa. However, the therapeutic mechanism of Li-ESW is not yet completely clear, and more research and exploration are still needed.

## Stem cell injection therapy

Stem cells can differentiate into different types of cells under the stimulation of complicated external environments and cytokines. They can be divided into totipotent stem cells, pluripotent stem cells, multipotent stem cells, and unipotent stem cells (57). Studies have shown that stem cells can also promote angiogenesis, tissue healing, and anti-apoptosis through paracrine action, which is also the theoretical basis of the therapy for ED (58). In the current scheme of stem cell therapy, it mainly includes mesenchymal stem cell (MSC), adipose tissue-derived stem cell (ADSC), urine-derived stem cell (UDSC), and muscle-derived stem cell (MDSC) (58, 59). In 2004, Bochinski et al. first discovered that injection of neural embryonic stem cells into the corpus cavernosa of male rats with neurogenic impotence could improve the erectile function (60). In the BCNI rat model, injection of autologous ADSC cells into the corpora cavernosa of rats can effectively prevent erectile dysfunction caused by cavernous nerve injury, enhance the ratio of smooth muscle over collagen content, and promote the neuronal nitric oxide synthase-positive nerve regeneration (61, 62). In rat model of erectile dysfunction, intracavernous injection of ADSCs can ameliorate ultrastructural damage and systemic oxidative stress states caused by chronic tobacco exposure or hyperlipidemia (63, 64). In addition, intracavernous injection of ADSCs-derived microtissues improves erectile function in STZ-induced diabetic rats *via* expressing vascular endothelial growth factor (VEGF), nerve growth factor (NGF), and tumor necrosis factor-stimulated gene-6 (TSG-6) (65). Furthermore, the exosomes secreted by stem cells have also been proved to have an effect on improving erectile dysfunction in rats. The study found that the injection of ADSC-derived exosomes into the cavernous body of rats can promote the growth of endothelial cells and smooth muscle cells, inhibit cell apoptosis, alleviate tissue hypoxia, and promote the recovery of erectile function in rats (66, 67).

Although many animal experiments have confirmed the effectiveness of stem cell therapy in treating ED, there are only a few clinical trials on stem cell therapy. In an open-label phase 1 clinical trial, 17 male patients with ED after radical prostatectomy were insensitive to PDE5I and ICI. The researchers injected autologous adipose-derived regenerative cells (ADRCs) into the corpora cavernosa of patients, the results showed that 73% of the patients recovered their erectile function within 3 months after treatment (68). According to animal experiments and a small number of clinical studies on stem cell therapy for ED, stem cell therapy is safe and reliable. Stem cell therapy has a broad prospect in the treatment of ED, but more clinical trials are still needed before clinical application.

## Conclusions and outlook

ED is a common disease in men and seriously affects the life quality of patients and their partners. Currently, oral PDE5I is the first-line treatment of ED which has the advantages of high safety and good effect. For patients with low response to PDE5I, other treatments include intracavernosal injection, hormonal treatment, vacuum erection device, and penile prosthesis implantation can also be alternative methods. In recent years, some new therapies like low-intensity extracorporeal shock wave and stem cell injection therapy proved to have exciting effect and can even reverse the organic damage of the corpora cavernosa. In fact, there are other advanced therapies, such as gene therapy (69), 3D-printed hydrogel scaffolds (70), and gene edited stem cells (71), they have all been shown to improve erectile function in animal experiments.

Despite these promising therapies are important directions for treatment of ED in the future, but only impressive animal studies proving the benefit. Large-scale, randomized, placebo-controlled studies are desperately needed for these novel therapeutics. In summary, ED is a complex disease associated with multiple risk factors, and effective therapy should be taken according to the etiology and individual conditions. The safety and efficacy of the promising therapies still need to be evaluated through a number of clinical trials with ethical support and fully informed consent.

## Author contributions

This mini review is contributed by all authors. XH and JX conceived and designed the study. CW and BW wrote the manuscript. CW, BW, PX, JX, and XH reviewed and edited the manuscript. All authors contributed to the article and approved the submitted version.
